# Supplementary material for: Effects of Spike Mutations in SARS-CoV-2 Variants of Concern on Human or Animal ACE2-Mediated Virus Entry and Neutralization
Source: bioRxiv. 2021 Aug 25:2021.08.25.457627. Preprint. [Version 1] doi: 10.1101/2021.08.25.457627 (PMC8404895; doi:10.1101/2021.08.25.457627)
Supplement: 1 [file NIHPP2021.08.25.457627V1-supplement-1.pdf]

bioRxiv preprint doi: https://doi.org/10.1101/2021.08.25.457627; this version posted August 25, 2021. The copyright holder for this preprint (which was not certified by peer review) is the author/funder, who has granted bioRxiv a license to display the preprint in perpetuity. It is made available under aCC-BY 4.0 International license.

Supplemental Table 1. Sources of human and animal ACE2 gene sequences.

**Species**	**Scientific name**	**Source**
Human	Homo sapiens	NP_068576.1
Cat	Felis catus	NP_001034545.1
Dog	Canis lupus	NP_001158732.1
Cattle	Bos taurus	NP_001019673.2
Horse	Equus caballus	XP_001490241.1
Camel – dromedary	Camelus dromedarius	XP_010991717.1
Hamster	Mesocricetus auratus	XP_005074266.1
Rabbit	Oryctolagus cuniculus	XP_002719891.1
European mink	Mustela lutreola biedermanni	MT560518
White tailed deer	Odocoileus virginianus	XP_020768965.1

bioRxiv preprint doi: https://doi.org/10.1101/2021.08.25.457627; this version posted August 25, 2021. The copyright holder for this preprint (which was not certified by peer review) is the author/funder, who has granted bioRxiv a license to display the preprint in perpetuity. It is made available under aCC-BY 4.0 International license.

Supplementary Table 2. Primers used for mutagenesis of the SARS-CoV-2 S gene.

Name	Sequences
SA2-D614G-F	5-TTGCTGTTCTTTATCAGGGTGTTAACTGCACAGAAGT-3
SA2-D614G-R	5-ACTTCTGTGCAGTTAACACCCTGATAAAGAACAGCAA-3
SA2-N501Y-F	5- GGTTTCCAACCCACTTATGGTGTTGGTTACCAAC-3
SA2-N501Y-R	5- GTTGGTAACCAACACCATAAGTGGGTTGGAAACC-3
SA2-E484K-F	5- CCTTGTAATGGTGTTAAAGGTTTTAATTGTTAC-3
SA2-E484K-R	5- GTAACAATTAAAACCTTTAACACCATTACAAGG-3
SA2-K417N-F	5- CCAGGGCAAACTGGAAACATTGCTGATTATAAT-3
SA2-K417N-R	5- ATTATAATCAGCAATGTTTCCAGTTTGCCCTGG3-
SA2-T478K-F	5' – ATCTATCAGGCCGGTAGCAAACCTTGTAATGGTGT-3
SA2-T478K-R	5' – ACACCATTACAAGGTTTGCTACCGGCCTGATAGAT-3
SA2-L452R-F	5-GGTTGGTGGTAATTATAATTACCGGTATAGATTGTTTAGGAAGTCTA-3
SA2-L452R-F	5-TAGACTTCCTAAACAATCTATACCGGTAATTATAATTACCACCAACC-3

bioRxiv preprint doi: https://doi.org/10.1101/2021.08.25.457627; this version posted August 25, 2021. The copyright holder for this preprint (which was not certified by peer review) is the author/funder, who has granted bioRxiv a license to display the preprint in perpetuity. It is made available under aCC-BY 4.0 International license.

Supplementary Table 3. SARS-CoV-2 strains used for *in vitro* replication kinetics and hamster infection experiments.

Strain	BEI number	Lineage	Clade	Spike Mutations	Variant
USA/WA1/2020	NR-52281	A	S	na	
USA/NY- PV08410/2020	NR-53514	B.1	GH	D614G	B.1
USA/CA CA_CDC_5574/2020	NR-54011	B.1.1.7	GR	D614G, N501Y	Alpha
South Africa/KRISP-K0053252020	NR-54009	B.1.351	GH	D614G, N501Y, E484K, K417N	Beta

na=not applicable
